# Transient localized surface plasmon induced by femtosecond interband excitation in gold nanoparticles

**DOI:** 10.1038/s41598-018-28909-6

**Published:** 2018-07-12

**Authors:** Xinping Zhang, Cuiying Huang, Meng Wang, Pei Huang, Xinkui He, Zhiyi Wei

**Affiliations:** 1 Institute of Information Photonics Technology and College of Applied Sciences, Beijng University of Technology, Beijing, 100124 P. R. China; 2 Institute of Physics, Beijing National Lab of Condensed Matter Physics, Chinese Academy of Science, Beijing, 100190 P. R. China

## Abstract

Localized surface plasmon resonance (LSPR) is essentially a collective oscillation of free electrons in nanostructured metals. Interband excitation may also produce conduction-band electrons above the Fermi level. However, a question here is whether these excited electrons can take part in plasmonic oscillation. To answer this question, femtosecond pump-probe measurements on gold nanoparticles were performed using interband excitation, where the pump pulse produced a large amount of electrons in the *sp*-conduction band and left holes in the *d*-band. Probing by transient absorption spectroscopy, we resolved an induced LSPR feature located at a red-shifted spectrum. This feature cannot be observed for a pumping photon energy lower than the threshold for interband transition. The commonly observed red-shift or broadening of LSPR spectrum due to electron-electron and electron-phonon scattering under strong optical excitation can be ruled out for understanding this feature by a comparison between the plasmonic dynamics at a pump above and below the interband-transition threshold. In particular, a “holding” time of about 1 ps was resolved for the interband-excitation-induced electrons to relax to the LSPR oscillation.

## Introduction

Ultrafast electronic relaxation performance is the basis for the application of localized surface plasmon resonance (LSPR) in optical switching devices based on nanostructured metals^1–5^. Strong optical excitation enhances electron-electron and electron-phonon scattering processes, leading to broadening and red-shift of the resonance spectrum of LSPR^6–10^. This is the main photophysics for the plasmonic optical switching effect. The related studies or applications are mainly based on the oscillation at optical frequencies of the free conduction-band electrons^11,12^, where the photon energy of the involved light beams is lower than the threshold for interband transition. When the excitation photon energy is above this threshold, interband transition will be induced, e.g. by electronic transition from *5d*-band to *6sp*-band in gold^13–15^. Such excitation definitely enhances electron density on the conduction band and will inevitably modulate the optical performance of the metallic nanostructures. However, due to high density of intrinsic conduction-band electrons, the interband-excitation-induced optical modulation can be detected only at high pump intensities. Additionally, plasmon electrons have a lifetime shorter than 100 fs. Thus, the weak modulation on the plasmonic dynamics by interband excitation could only be investigated by transient absorption spectroscopy using ultrashort pulses with high peak intensities^16–20^. Although spectroscopic response of interband transition has been investigated in different nanostructured metals, there is still a question to be clarified, if the conduction-band electrons induced by interband excitation behave in the same manner as the intrinsic plasmonic electrons. In this work, we investigate how the interband-excitation-induced conduction-band electrons influence the plasmonic dynamics by femtosecond pump-probe detection. In particular, we supply evidence that these excited electrons are directly involved in plasmonic oscillations.

## Interband-excitation of plasmonic electrons in gold nanoparticles

**Figure 1:**
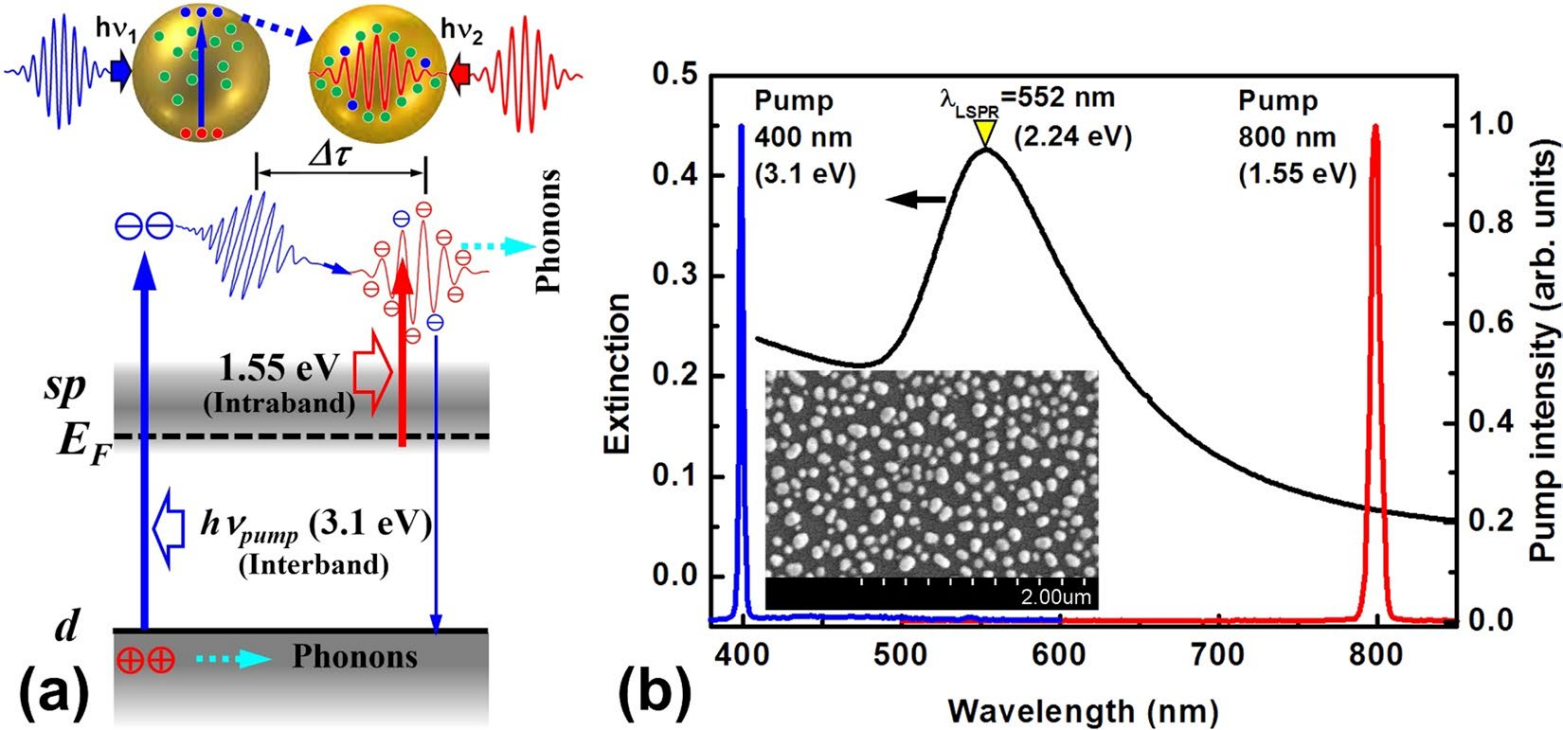
(**a**) The schematic illustration of the interband (at 3.1 eV) and intraband (at 1.55 eV) excitation and the relaxation of the excited conduction-band electrons into plasmonic oscillation. Δτ measures the time delay for the interband excitation produced electrons to “join” the plasmonic oscillation process. Dashed arrows in light blue illustrate energy transfer from electrons/holes to lattices through phonon population, as relaxation pathways. (**b**) Optical extinction spectrum (black) measured on the AuNPs shown in the inset by the SEM image and the pump spectrum centered at 400 (blue) and 800 nm (red) in the transient absorption measurement.

Figure 1(a) shows schematically the possible mechanisms that are involved in the interband excitation and the subsequent plasmonic oscillation in gold nanoparticles (AuNPs). For gold, the threshold energy for interband transition is about 2.38 eV^21^. Therefore, the excitation at 3.1 eV (400 nm) induced excitation of electronic transition from 5*d* band to the hybridized 6*sp* band in gold, leaving holes in 5*d* band. This results in a transient electron population or a transient increase in the electron density in the conduction band. Thus, we propose in this work the following mechanisms after the interband excitation: (1) The interband-excitation produced conduction-band electrons can also be involved in the plasmonic dynamics. The excess energy in the holes is released mainly to the lattices, as well as to the conduction-band electrons^21^. (2) There exists a time delay before the interband-excitation-produced electrons relax and take part in the plasmonic oscillation. An intraband relaxation and an adaptive phase/frequency adjustment are needed for the newly generated electrons to take part in the plasmonic oscillation process, which leads to a time delay of the induced LSPR, as indicated by Δτ in Fig. 1(a). (3) The new generation or the increased density of the conduction-band electrons will lead to stronger interaction between the plasmonic electrons. The scattering between these electrons slows down the speed of plasmonic oscillation. As a result, the resonance frequency will be reduced, resulting in a red-shifted resonance spectrum. (4) Due to the excess energies of both the hot electrons and the holes, the “heating” of the lattices becomes stronger, resulting in much enhanced and extended phonon relaxation process^10,21^. The heating of the lattice is mainly an interaction between plasmonic charge carriers and phonons, where energy is transferred from hot electrons/holes to the surrounding lattices. This not only extends the time scale of electron-phonon interaction, but also consequently enhances the phonon-scattering process. The above mechanisms were verified by the experimental results presented in section 3, which could not be observed for a pump photon energy (e.g. at 1.55 eV, as shown in Fig. 1(a)) lower than the threshold for the interband transition.

Figure 1(b) shows the optical extinction spectrum of a random matrix of AuNPs with their scanning electron microscopic (SEM) image presented in the inset. The AuNPs were fabricated by annealing a thin-film of colloidal gold nanoparticles at 400 °C, which was prepared by spin-coating the colloidal solution with a concentration of 100 mg/mL onto a silica substrate. The colloidal gold nanoparticles have a diameter ranging from 5 to 10 nm and were covered with ligands to be suspended with excellent dispersity in xylene. The LSPR spectrum is peaked at about 552 nm. The off-resonance excitation in the femtosecond pump-probe measurement is located at 800 and 400 nm, which was the output from a Ti:sapphire amplifier before and after the frequency doubling scheme, as plotted by the red and blue spectrum, respectively, in Fig. 1(b). The excitation at 400 nm (blue spectrum) induced interband transitions in the AuNPs and the 800-nm excitation supplied a direct comparison for resolving the interband excitation induced plasmonic effects. The inset SEM image provides a rough evaluation on the mean size and random distribution of the AuNPs.

## Evolution dynamics of the interband-excitation-induced LSPR

Pump-probe using 150-fs pulses at 400 nm and 800 nm with a repetition rate of 1 kHz was performed on the matrix of AuNPs shown in the inset of Fig. 1(b), where the probe pulses were generated by focusing a small portion of the 800-nm pulses into heavy water in a 3-mm-thick cuvette^7,12^. The spectral range of the probe pulses extends from about 300 to 1200 nm, however, our investigation was focused on the range from 400 to 800 nm, which covered the LSPR spectrum. The 800-nm pulses produced by a Ti:sapphire amplifier from Coherent Ltd. were frequency-doubled by a BBO crystal to produce 400-nm pump pulses. The pump fluence of about 300 μJ/cm^2^ was employed for the 800-nm pumping and that lower than 50 μJ/cm^2^ for the 400-nm pumping. These pump fluence values ensured reliable transient absorption data without damaging the gold nanostructures, which can be verified by the reproduced TA spectra/dynamics after multiple cycles of measurements.

**Figure 2:**
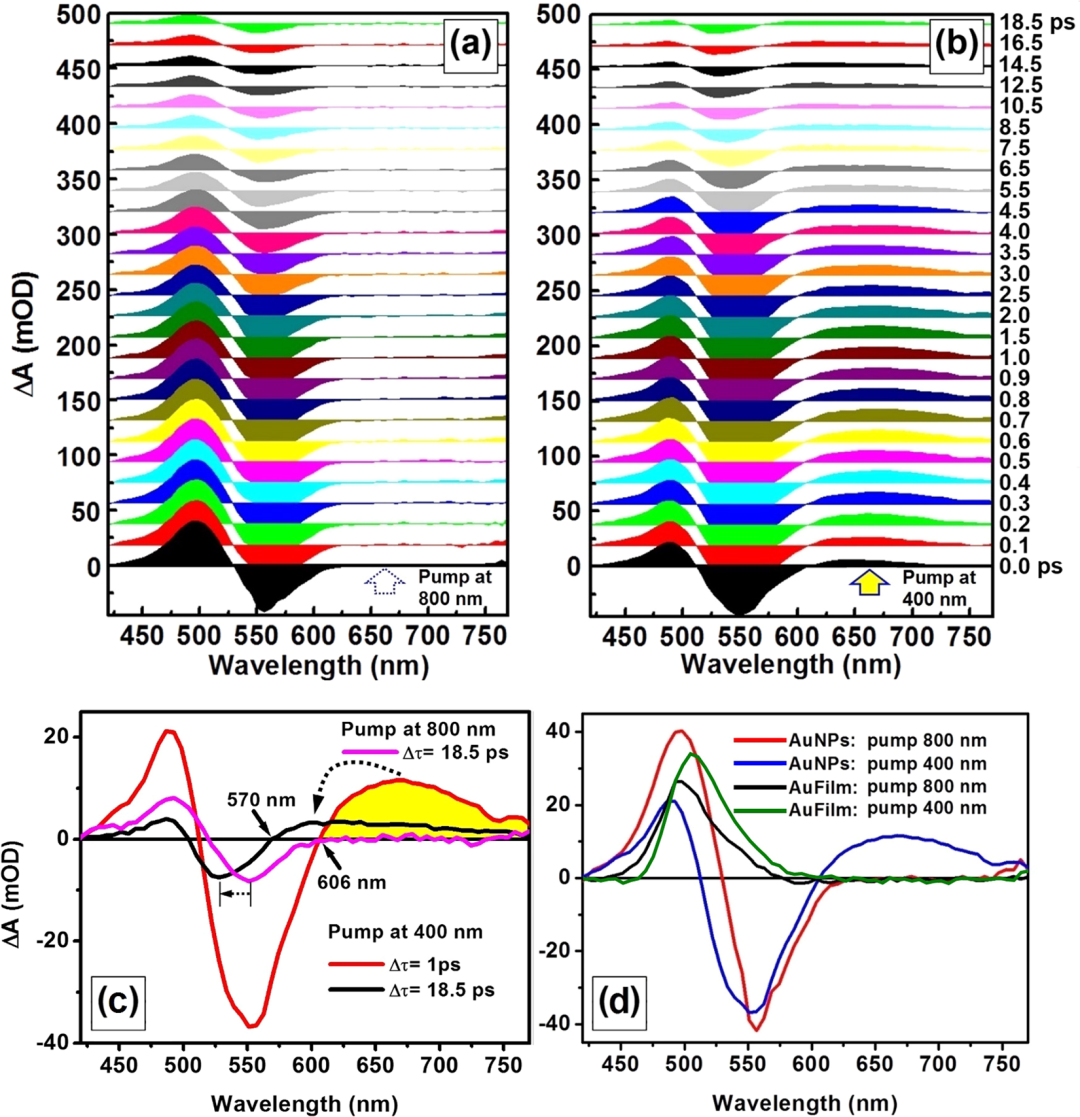
(**a**,**b**) Transient absorption spectra at a time delay increased from 0 to 18.5 ps for a pump wavelength of 800 and 400 nm, respectively. (**c**) Comparison between the TA spectra measured on the gold nanoparticles at a pump wavelength of 400 nm and a time delay of 1 ps (red) and 18.5 ps (black) and at a pump wavelength of 800 nm and a time delay of 18.5 ps (magenta, dashed). (**d**) Comparison between the TA spectra measured on gold nanoparticles at a pump wavelength of 800 nm (red) and 400 nm (blue) and on a gold film at a pump wavelength of 800 nm (black) and 400 nm (green).

Figure 2(a,b) show the TA spectra ranging from 420 to 770 nm for pumping at 800 and 400 nm, respectively, as the delay between the pump and the probe pulse was increased from 0 to 18.5 ps. The common features of these two groups of data lie in the positive spectra peaked at about 490 nm and a negative dip at about 550 nm. However, a special feature can be observed for the 400-nm pumped TA spectra, which is a positive transient spectrum extending from shorter than 600 nm to longer than 770 nm. However, such feature is not observable for the TA spectra for 800-nm pumping. The positive feature is shown into more details in Fig. 2(c), as indicated by the yellow-filled spectrum. The TA spectra for a pump wavelength of 400 nm at a delay of 1 ps and 18.5 ps, as well as that for a pump wavelength of 800 nm at a delay of 18.5 ps, are presented in Fig. 2(c). Clearly, for pumping at 400 nm, with increasing the time delay there is not only a reduction in the amplitude of the TA signals, but also a retreating of the negative or forward-stepping of the positive spectral features to shorter wavelengths, so that the crossing point of the spectrum through the zero TA line shifted from 606 to 570 nm. This confirms a transient *LSPR*-like spectrum peaked at about 670 nm due to interband excitation, which covers a broad spectral band from shorter than 570 nm to longer than 770 nm and overlaps largely the bleached intrinsic LSPR spectrum with negative ΔA (red curve in Fig. 2(c)). We define this feature as interband-excitation-induced plasmon and simplify it as *EIP*. The above process can be more clearly understood by considering that there is a spectral overlap between the bleaching of the intrinsic plasmon resonance, which exhibits a negative TA spectrum peaked at a shorter wavelength of about 552 nm, and the interband-excitation induced LSPR, which exhibits a positive TA spectrum peaked at a longer wavelength of about 670 nm. The bleaching of the intrinsic plasmon decays faster than the *EIP*, therefore, the positive LSPR overtakes the bleaching signal gradually with increasing the delay time. In contrast, we use an abbreviation of *IP* to designate the intrinsic plasmon performance of the AuNPs. From Fig. 2(c) we can understand that the bleaching of the *IP* curve due to pumping at 400 nm, as shown by the red spectrum, was modulated by the *EIP* and evolved as a competition between these two processes.

The positive spectral feature peaked at about 490 nm is attributed to the modulation around the threshold point for interband transitions, where electron-hole pairs have been produced due to the excitation from the *d*-band to the *sp*-conduction-band. At the threshold point, a strong “reduction” in the optical reflection is generally observed in the steady-state spectroscopic performance, which is typical for noble metals. However, strong optical excitation may “bleach” such effect, where the “reduction” in optical reflection will become smaller, leading to a transient enhancement of reflection. This explains why a positive TA signal is always observed in the transmissive pump-probe measurements on different nanostructures of noble metals, which also applies to other pump wavelengths. In Fig. 2(d), we show the TA spectra measured on AuNPs using a pump at 400 nm, on the AuNPs using a pump at 800 nm, on a 40-nm-thick gold film (AuFilm) using a pump at 400 nm, and on a 40-nm-thick AuFilm using a pump at 800 nm^12^ by the blue, red, black, and green curves, respectively, where the AuFilm was produced by thermal evaporation. All of the spectra in Fig. 2(d) show positive peaks at about 490 nm, which was clearly based on the same bleaching mechanism. The gold film exhibits very similar performance for 400- and 800-nm pumping. The transient plasmon peaked at 670 nm is only observed for the 400-nm pumping on the AuNPs.

The negative spectral feature observable for both 400-nm- and 800-nm-pumping resulted mainly from the bleaching of the optical extinction by intrinsic plasmon (*IP*) of the AuNPs. However, there is a blue shift as large as 24 nm for these two negative features at a same time delay of 18.5 ps, as indicated by the leftward arrow from 551 to 527 nm in Fig. 2(c). One reason is that the *EIP* feature appeared as a positive spectrum, which pushed the rising edge of the negative spectrum to shorter wavelengths when pumping at 400 nm. This also implies broad-band performance of the *EIP* resonance spectrum. Another reason is the bleaching of the interband absorption for wavelengths shorter than 520 nm or for photon energies smaller than 2.38 eV, which becomes more prominent at longer time delays. Interband transition takes place at wavelengths shorter than the threshold at about 520 nm. Pumping at 400 nm excited strong interband transition, leading to depopulation of the *d*-band and bleaching of the interband absorption. This bleaching mechanism should be distinguished from that at the turning point between inter- and intra-band transitions, which was observed as a positive TA feature. However, they have an overlap in the spectrum, which has also modulated the TA dynamics.

**Figure 3:**
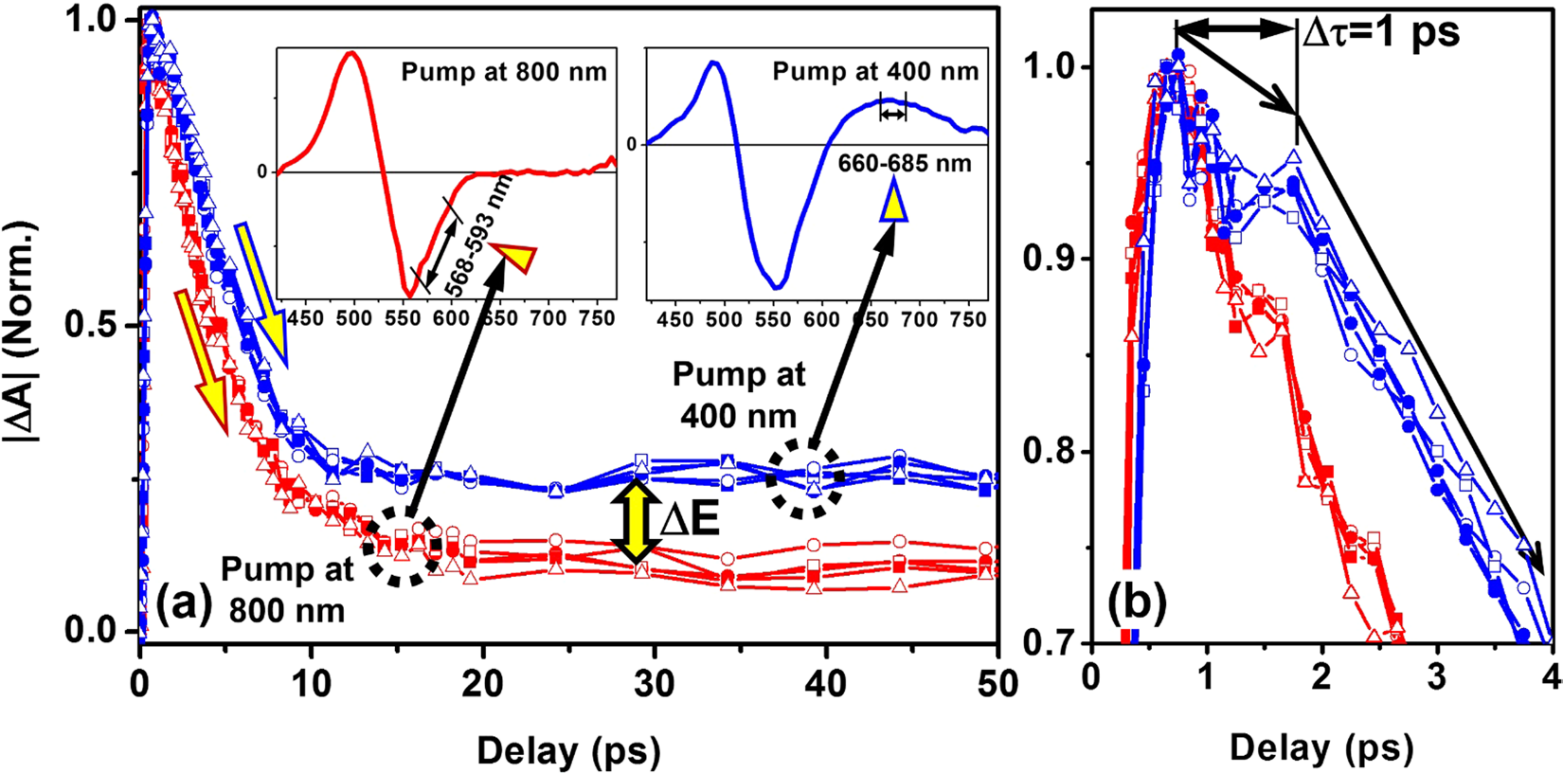
(**a**) Comparison between the bleaching dynamics of the IP when pumping at 800 nm (red, opposite values of ΔA) and probing in a spectral range from 568 to 593 nm and the relaxation dynamics of the EIP when pumping at 400 nm (blue) and probing in a spectral range from 660 to 685 nm, as indicated using triangles in the inset TA spectra. (**b**) An enlarged observation for a time delay from 0 to 4 ps, showing the evolution dynamics with different relaxation rates and a 1-ps holding time.

For understanding more clearly the *EIP* feature peaked at about 670 nm, we need to compare TA dynamics of the plasmons at a pump photon energy higher and lower than the interband-transition threshold. Since the *EIP* feature cannot be observed at a pump photon energy lower than the interband transition threshold, we compared the TA dynamics of the *IP* bleaching process for a pump at 800 nm (1.55 eV) with the *EIP* excited by a pump at 400 nm (3.1 eV), as shown in Fig. 3. However, the bleached *IP* corresponds to negative TA dynamics, for convenience, we made a comparison between the absolute values of transient absorption (|ΔA|) in Fig. 3.

In Fig. 3(a), the blue curves show the measurements of the TA dynamics for a pump wavelength of 400 nm, and the red ones show those using a pump wavelength of 800 nm, which correspond to the *EIP*-relaxation and *IP*-bleaching dynamics, respectively. The *IP*-bleaching dynamics at different wavelengths in a spectral band from 568 to 593 nm (left panel of the inset) are presented for pumping at 800 nm, and the *EIP*-relaxation dynamics are shown in the spectral range from 660 to 685 nm (right panel of the inset) for pumping at 400 nm, where all of the curves have been normalized. The blue and the red curves exhibit consistent relaxation dynamics, respectively. Compared with the red dynamic curves, the blue ones “hold” around the peak TA dynamics with very small decay within the first 1 ps, which is followed by a faster relaxation, as shown in Fig. 3(b) by a more detailed observation in the time-delay range of 0~4 ps. After the “holding” stage, the blue curves decay at nearly the same rate as the red, as indicated by the parallel downward blue and red arrows in Fig. 3(a). This verifies the plasmonic oscillation performance of the interband excited conduction-band electrons. In the third stage, both the blue and red curves relax very slowly, however, the TA signals are much larger in amplitude at a 400-nm pump than at 800 nm, we specified the difference as *ΔE*. This verifies the diffusion of the excess energies of the interband-excitation induced holes in the *d*-band and the hot electrons to the lattices, enhancing the electron-phonon interaction process.

We present five dynamic curves in the blue at 568, 573, 578, 583, and 593 nm for the spectral range from 568 to 593 nm for pumping at 800 nm, as specified in the inset on the left hand in Fig. 3(a). This intends to demonstrate the consistence of the physics for different wavelengths in this featured spectral band. Similarly, we also included 5 dynamic curves at 660, 665, 670, 675, and 685 nm to show the consistent mechanisms at different wavelengths in the spectral range from 660 to 685 nm for pumping at 400 nm. These multiple dynamics with the same color have almost the same decay dynamics, implying respectively consistent physics within these two spectral bands.

The 1-ps “holding time” is the most important modulation on the TA dynamics by the interband excitation at 400 nm. Here, we use “holding time” to describe the transient slow-down process of the TA dynamics due to the sudden generation of the conduction-band electrons by interband excitation. It would take some time for this portion of electrons to relax in an intra-band process and adjust themselves to adapt in phase and frequency to the plasmonic oscillation process. Furthermore, the interband excitation increased dramatically the electron density on the conduction band, which consequently enhanced the electron-electron and electron-phonon scattering and energy-transfer processes. As a result, the optical extinction was enhanced and delayed with respect to the intrinsic plasmonic processes in the AuNPs at a red-shifted spectrum.

After the first stage, the relaxation process was dominated by the plasmonic dynamics, where all of the electrons took part in the collective oscillation defined by the geometric and dielectric parameters of the gold nanostructures. In this stage, the dynamics based on interband-excitation has nearly the same response time as the intrinsic plasmons. This is why we observed a similar decay rate as indicated by the downward arrows. However, the decay dynamics took more than 5 ps, implying an apparent electron-phonon interaction process. This can be understood by considering the pump pulse duration as long as 150 fs, which is not short enough to resolve the pure electronic process.

Additionally, the interband excitation produced much excess energy in the conduction-band electrons and in the *d*-band holes. These excess energies were mainly released to the lattices, resulting in much enhancement of the long-lived thermal effects or the phonon-phonon interaction process, as indicated roughly by the difference of ΔE in Fig. 3.

In Fig. 3, we have chosen two different spectral bands to compare the TA dynamics. The reason is that these TA dynamics can stably display the spectroscopic performance of the studied features of *EIP* and *IP*, although values of the TA data have different signs. In fact, the transient LSPR of *EIP* has a broad-band influence on its “neighboring” spectrum, which extends from shorter than 570 nm to longer than 770 nm, according to above analysis. There is a spectral overlap between the *EIP* and the bleaching of the *IP*. However, the TA signals for *EIP* and for the bleached *IP* have different response times. There will be a disturbance to the TA signal if we choose a spectral range covering both modes. The negative TA spectra in Fig. 2(b) resulted from the bleaching of *IP* was already modulated by the positive *EIP*. Therefore, it is not reasonable to use the negative TA signal to evaluate the dynamics of *EIP* and we have chosen a spectral range from 660 to 685 nm, which is separated far enough from the peak spectrum of the *IP*.

**Figure 4:**
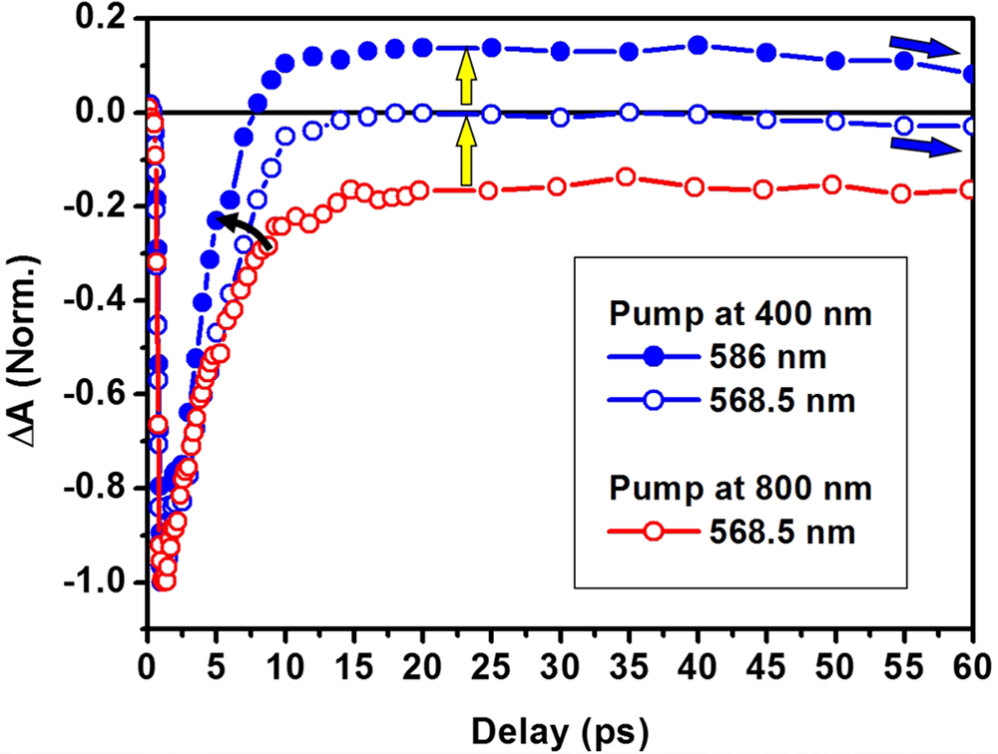
TA dynamics measured at 568.5 nm for pumping at 400 (blue open circles) and 800 nm (red open circles) with the TA dynamics (blue solid circles) at 568 nm for a pump wavelength of 400 nm presented for comparison.

Further analyses are shown in Fig. 4, where we show three dynamic curves with two at 568.5 nm for both 400- and 800-nm pumping and one at 586 nm for 400-nm pumping. Due to the influence by the transient *EIP* with a positive TA spectrum and delayed dynamics, the bleaching signal recovered much faster to zero for 400-nm pumping than for 800-nm pumping. The positive TA by *EIP* counteracted the negative bleaching signal, accelerating the recovery of transient absorption process. The “acceleration” effect becomes even more obvious at 586 nm for 400-nm pumping, as shown by the blue dynamic curve with filled circles in Fig. 4. The TA signal for the bleaching recovered to zero at a delay of only 7 ps before becoming positive. This verifies the broad-band and time-delayed features of *EIP* with respect to *IP*. However, the TA dynamics drops again after the strong modulation by the transient *EIP*, as indicated by the blue arrows to the right. This is a recovery back to the strong and long-lived phonon-involved relaxation process. In particular, the excess energy due to the generation of *d*-band holes after interband excitation needs to be released to the lattices.

## Conclusions

We observed a transient plasmon feature induced by interband excitation of gold nanoparticles, which is located in a red-shifted spectrum with respect to the intrinsic mode. Such feature was not observed when the excitation photon energy is lower than the threshold for interband transition, confirming that the new plasmon feature resulted from modification on the conduction-band electron dynamics by interband-excitation. The increased electron density enhanced electron-electron scattering processes and slowed down the damping rate of the plasmonic electrons. This is equivalent to a longer-distance travelling of the electrons within the same nanostructures under optical electric field. Thus, the resonance oscillation requires interaction with an optical electric field with a longer wavelength, leading to red-shift of LSPR spectrum. Furthermore, a holding time of about 1 ps was resolved for the interband electrons to relax into plasmonic oscillation, which was observed as a much slower decay dynamics than the intrinsic plasmon.
